# Supplementary material for: Deletion of wfs1 Impairs Oligodendrocyte Precursor Cells Dorsal Distribution and Myelination Through the wfs1-hmgcs1 Axis in Zebrafish
Source: Biology (Basel). 2026 Aug 16;15(16):1402. doi: 10.3390/biology15161402 (PMC13510151; doi:10.3390/biology15161402)
Supplement: Supplementary file 1 [file biology-15-01402-s001.zip › File S1-Transcriptome sequencing data and differentially expressed genes (DEGs) in wfs1-- zebrafish at 5 dpf/2.KEGG/wfs1KOvsWT/GSEA/WT/GAP_JUNCTION/GAP_JUNCTION(DRE04540).html]

Details for gene set GAP\_JUNCTION(DRE04540)[GSEA]

|  || Dataset | deg.sample |
| Phenotype | sample.cls |
| Upregulated in class | WT |
| GeneSet | GAP\_JUNCTION(DRE04540) |
| Enrichment Score (ES) | -0.23640709 |
| Normalized Enrichment Score (NES) | -1.1095403 |
| Nominal p-value | 0.25203252 |
| FDR q-value | 0.53096086 |
| FWER p-Value | 1.0 |
Table: GSEA Results Summary

  

Fig 1: Enrichment plot: GAP\_JUNCTION(DRE04540)      
 Profile of the Running ES Score & Positions of GeneSet Members on the Rank Ordered List

  

| PROBE | DESCRIPTION (from dataset) | GENE SYMBOL | GENE\_TITLE | RANK IN GENE LIST | RANK METRIC SCORE | RUNNING ES | CORE ENRICHMENT || 1 | 323509\_1 | gnaia |  |  | 312 | 3.596 | 0.0279 | No |
| 2 | 100006675 | prkcba |  |  | 711 | 2.563 | 0.0422 | No |
| 3 | 796490\_2 | pdgfbb |  |  | 882 | 2.259 | 0.0605 | No |
| 4 | 568726 | adcy7 |  |  | 1220 | 1.881 | 0.0696 | No |
| 5 | 103909601 | LOC103909601 |  |  | 1276 | 1.830 | 0.0871 | No |
| 6 | 560807 | adcy6b |  |  | 2040 | 1.353 | 0.0772 | No |
| 7 | 431777 | tuba7l |  |  | 2071 | 1.338 | 0.0903 | No |
| 8 | 373080 | tuba1b |  |  | 2072 | 1.337 | 0.1043 | No |
| 9 | 563724 | adcy2a |  |  | 2111 | 1.321 | 0.1170 | No |
| 10 | 559806 | htr2ab |  |  | 2342 | 1.232 | 0.1227 | No |
| 11 | 560564 | pdgfc |  |  | 2476 | 1.180 | 0.1308 | No |
| 12 | 100125912\_1 | prkg2 |  |  | 3006 | 1.034 | 0.1250 | No |
| 13 | 559529 | pdgfrb |  |  | 3029 | 1.028 | 0.1351 | No |
| 14 | 573216 | tuba1a |  |  | 3240 | 0.984 | 0.1388 | No |
| 15 | 103909601\_1 | LOC103909601 |  |  | 3250 | 0.982 | 0.1488 | No |
| 16 | 567611 | itpr1a |  |  | 3360 | 0.962 | 0.1555 | No |
| 17 | 570652 | adcy6a |  |  | 3581 | 0.914 | 0.1581 | No |
| 18 | 557194\_1 | adrb1 |  |  | 3690 | 0.895 | 0.1641 | No |
| 19 | 337223 | tuba8l |  |  | 3910 | 0.846 | 0.1661 | No |
| 20 | 326020 | si:dkey-121j17.5 |  |  | 4627 | 0.731 | 0.1512 | No |
| 21 | 100537538 | LOC100537538 |  |  | 4897 | 0.694 | 0.1500 | No |
| 22 | 564719 | adcy3b |  |  | 4985 | 0.683 | 0.1544 | No |
| 23 | 100007679 | map2k5 |  |  | 5574 | 0.629 | 0.1425 | No |
| 24 | 565638 | LOC565638 |  |  | 6141 | 0.573 | 0.1306 | No |
| 25 | 393998 | prkacba |  |  | 6234 | 0.564 | 0.1337 | No |
| 26 | 558321 | map3k2 |  |  | 6562 | 0.532 | 0.1289 | No |
| 27 | 541323 | mapk7 |  |  | 6744 | 0.515 | 0.1286 | No |
| 28 | 378718 | drd2l |  |  | 6847 | 0.505 | 0.1307 | No |
| 29 | 325084 | src |  |  | 6875 | 0.502 | 0.1351 | No |
| 30 | 378444\_2 | gjd2b |  |  | 6883 | 0.502 | 0.1402 | No |
| 31 | 493613 | gna11b |  |  | 7043 | 0.488 | 0.1403 | No |
| 32 | 792024 | si:ch73-199e17.1 |  |  | 7117 | 0.481 | 0.1430 | No |
| 33 | 336421\_1 | gnai2a |  |  | 7177 | 0.475 | 0.1461 | No |
| 34 | 572372\_1 | gjd1a |  |  | 7273 | 0.468 | 0.1481 | No |
| 35 | 393154 | tuba8l4 |  |  | 7289 | 0.467 | 0.1525 | No |
| 36 | 100331216 | LOC100331216 |  |  | 7737 | 0.440 | 0.1430 | No |
| 37 | 550420\_1 | gucy1a3 |  |  | 7860 | 0.434 | 0.1437 | No |
| 38 | 562585 | itpr2 |  |  | 8204 | 0.415 | 0.1373 | No |
| 39 | 560807\_1 | adcy6b |  |  | 8280 | 0.414 | 0.1392 | No |
| 40 | 767790 | pdgfab |  |  | 8507 | 0.402 | 0.1363 | No |
| 41 | 550420 | gucy1a3 |  |  | 8949 | 0.371 | 0.1263 | No |
| 42 | 100332913 | grm5b |  |  | 9058 | 0.363 | 0.1267 | No |
| 43 | 571825 | adcy3a |  |  | 9521 | 0.332 | 0.1157 | No |
| 44 | 393664 | tuba8l2 |  |  | 9767 | 0.318 | 0.1113 | No |
| 45 | 378845 | tjp1a |  |  | 9932 | 0.308 | 0.1093 | No |
| 46 | 327377 | tuba4l |  |  | 9990 | 0.304 | 0.1107 | No |
| 47 | 100535718 | pdgfd |  |  | 10271 | 0.286 | 0.1049 | No |
| 48 | 100005880 | cx36.7 |  |  | 10308 | 0.284 | 0.1068 | No |
| 49 | 641421 | tubb2b |  |  | 10342 | 0.282 | 0.1087 | No |
| 50 | 386856 | pdgfra |  |  | 10436 | 0.277 | 0.1086 | No |
| 51 | 322106 | csnk1da |  |  | 10739 | 0.259 | 0.1018 | No |
| 52 | 393228\_1 | zgc:55558 |  |  | 10754 | 0.257 | 0.1041 | No |
| 53 | 563181 | map2k2a |  |  | 10792 | 0.256 | 0.1056 | No |
| 54 | 557191 | gucy1b2 |  |  | 10854 | 0.252 | 0.1063 | No |
| 55 | 497384 | prkca |  |  | 10900 | 0.249 | 0.1075 | No |
| 56 | 100333538 | LOC100333538 |  |  | 11027 | 0.242 | 0.1061 | No |
| 57 | 406811\_2 | zgc:55461 |  |  | 11028 | 0.242 | 0.1086 | No |
| 58 | 560410 | si:ch211-132f19.7 |  |  | 11057 | 0.241 | 0.1103 | No |
| 59 | 407700 | plcb4 |  |  | 11151 | 0.236 | 0.1098 | No |
| 60 | 100149080 | LOC100149080 |  |  | 11197 | 0.233 | 0.1108 | No |
| 61 | 566430 | prkg1b |  |  | 11541 | 0.214 | 0.1023 | No |
| 62 | 565459 | tjp1b |  |  | 11806 | 0.201 | 0.0961 | No |
| 63 | 386701 | tubb5 |  |  | 11807 | 0.201 | 0.0982 | No |
| 64 | 556339\_1 | LOC556339 |  |  | 11868 | 0.197 | 0.0984 | No |
| 65 | 393953 | prkcbb |  |  | 11901 | 0.196 | 0.0994 | No |
| 66 | 560719 | si:dkey-206f10.1 |  |  | 11948 | 0.193 | 0.1000 | No |
| 67 | 100003514 | prkcg |  |  | 12176 | 0.181 | 0.0947 | No |
| 68 | 406533 | csnk1db |  |  | 12430 | 0.168 | 0.0885 | No |
| 69 | 792634 | drd1a |  |  | 12455 | 0.167 | 0.0895 | No |
| 70 | 557353 | gnas |  |  | 12479 | 0.165 | 0.0905 | No |
| 71 | 336681 | tubb4b |  |  | 13139 | 0.133 | 0.0711 | No |
| 72 | 570108 | gnaq |  |  | 13257 | 0.128 | 0.0688 | No |
| 73 | 556339 | LOC556339 |  |  | 13325 | 0.124 | 0.0680 | No |
| 74 | 80973 | cdk1 |  |  | 13334 | 0.124 | 0.0690 | No |
| 75 | 378444\_1 | gjd2b |  |  | 13623 | 0.110 | 0.0611 | No |
| 76 | 378719 | drd2b |  |  | 13665 | 0.108 | 0.0609 | No |
| 77 | 557902 | adcy2b |  |  | 13739 | 0.105 | 0.0597 | No |
| 78 | 563147 | prkacbb |  |  | 13940 | 0.095 | 0.0544 | No |
| 79 | 561670 | sos2 |  |  | 14111 | 0.087 | 0.0500 | No |
| 80 | 100149388 | itpr1b |  |  | 14261 | 0.080 | 0.0461 | No |
| 81 | 557026 | adcy1a |  |  | 14359 | 0.076 | 0.0439 | No |
| 82 | 564064 | prkacaa |  |  | 14469 | 0.071 | 0.0412 | No |
| 83 | 100001532 | plcb3 |  |  | 14510 | 0.069 | 0.0406 | No |
| 84 | 100006888 | gnai3 |  |  | 15454 | 0.024 | 0.0112 | No |
| 85 | 751784 | htr2b |  |  | 15535 | 0.020 | 0.0089 | No |
| 86 | 30380 | nras |  |  | 15635 | 0.015 | 0.0059 | No |
| 87 | 445164 | tuba8l3 |  |  | 15740 | 0.009 | 0.0027 | No |
| 88 | 563953\_1 | gna11a |  |  | 15848 | 0.005 | -0.0006 | No |
| 89 | 406308 | grb2b |  |  | 15876 | 0.003 | -0.0014 | No |
| 90 | 100334937 | LOC100334937 |  |  | 16047 | -0.003 | -0.0067 | No |
| 91 | 568126 | drd1b |  |  | 16404 | -0.014 | -0.0178 | No |
| 92 | 378478 | egfra |  |  | 16429 | -0.015 | -0.0184 | No |
| 93 | 368461 | lpar1 |  |  | 16539 | -0.018 | -0.0216 | No |
| 94 | 282557 | drd2a |  |  | 16622 | -0.021 | -0.0240 | No |
| 95 | 445076 | prkacab |  |  | 17201 | -0.041 | -0.0418 | No |
| 96 | 30236 | cx43 |  |  | 17236 | -0.042 | -0.0424 | No |
| 97 | 569499 | adcy1b |  |  | 17365 | -0.048 | -0.0460 | No |
| 98 | 767790\_1 | pdgfab |  |  | 18004 | -0.076 | -0.0653 | No |
| 99 | 550458 | tubb6 |  |  | 18472 | -0.097 | -0.0790 | No |
| 100 | 100333702 | adcy9 |  |  | 18501 | -0.099 | -0.0788 | No |
| 101 | 562619 | adcy5 |  |  | 18631 | -0.105 | -0.0818 | No |
| 102 | 336421 | gnai2a |  |  | 18634 | -0.105 | -0.0807 | No |
| 103 | 327650 | gnai2b |  |  | 19453 | -0.142 | -0.1050 | No |
| 104 | 399480 | mapk3 |  |  | 19514 | -0.144 | -0.1054 | No |
| 105 | 406728 | map2k1 |  |  | 19540 | -0.145 | -0.1047 | No |
| 106 | 557194 | adrb1 |  |  | 19650 | -0.150 | -0.1065 | No |
| 107 | 100150304 | gucy1b3 |  |  | 19772 | -0.156 | -0.1087 | No |
| 108 | 798657\_1 | si:ch211-79m20.1 |  |  | 19845 | -0.159 | -0.1093 | No |
| 109 | 563953 | gna11a |  |  | 19873 | -0.160 | -0.1085 | No |
| 110 | 557109 | raf1b |  |  | 20203 | -0.175 | -0.1170 | No |
| 111 | 569376 | plcb2 |  |  | 20503 | -0.188 | -0.1245 | No |
| 112 | 554127 | tubb2 |  |  | 20659 | -0.196 | -0.1273 | No |
| 113 | 100007679\_1 | map2k5 |  |  | 20661 | -0.197 | -0.1253 | No |
| 114 | 493609\_1 | grb2a |  |  | 20689 | -0.198 | -0.1240 | No |
| 115 | 335798 | zgc:65894 |  |  | 21348 | -0.234 | -0.1423 | No |
| 116 | 378719\_1 | drd2b |  |  | 22017 | -0.268 | -0.1606 | No |
| 117 | 406303 | tuba2 |  |  | 22113 | -0.274 | -0.1607 | No |
| 118 | 393946 | gnai1 |  |  | 22152 | -0.276 | -0.1590 | No |
| 119 | 100148976\_1 | adcy8 |  |  | 22476 | -0.295 | -0.1661 | No |
| 120 | 555576 | grm1a |  |  | 23212 | -0.342 | -0.1857 | No |
| 121 | 360144\_1 | mapk1 |  |  | 23446 | -0.357 | -0.1893 | No |
| 122 | 795591 | si:ch211-114n24.6 |  |  | 23557 | -0.364 | -0.1889 | No |
| 123 | 567611\_1 | itpr1a |  |  | 24145 | -0.403 | -0.2032 | No |
| 124 | 100150246\_1 | grm1b |  |  | 24426 | -0.416 | -0.2077 | No |
| 125 | 100002225 | map2k2b |  |  | 24736 | -0.433 | -0.2129 | No |
| 126 | novel.939 | - |  |  | 24769 | -0.436 | -0.2093 | No |
| 127 | 796490 | pdgfbb |  |  | 25120 | -0.455 | -0.2156 | No |
| 128 | 101884360 | LOC101884360 |  |  | 25255 | -0.464 | -0.2149 | No |
| 129 | 570652\_2 | adcy6a |  |  | 25465 | -0.481 | -0.2165 | No |
| 130 | 100148976 | adcy8 |  |  | 25826 | -0.513 | -0.2224 | No |
| 131 | 30716 | raf1a |  |  | 25882 | -0.518 | -0.2187 | No |
| 132 | 394005 | prkg1a |  |  | 26027 | -0.531 | -0.2177 | No |
| 133 | 550286 | hrasa |  |  | 26472 | -0.575 | -0.2256 | No |
| 134 | 100333967 | si:ch73-374l24.1 |  |  | 26564 | -0.584 | -0.2224 | No |
| 135 | 100000981 | htr2cl1 |  |  | 26669 | -0.595 | -0.2194 | No |
| 136 | 794666\_1 | itpr3 |  |  | 26950 | -0.625 | -0.2217 | No |
| 137 | 560808 | htr2aa |  |  | 26956 | -0.625 | -0.2153 | No |
| 138 | 553656 | hrasb |  |  | 27554 | -0.680 | -0.2270 | No |
| 139 | 497384\_1 | prkca |  |  | 27854 | -0.723 | -0.2288 | Yes |
| 140 | 798657 | si:ch211-79m20.1 |  |  | 28060 | -0.751 | -0.2274 | Yes |
| 141 | 403045\_1 | egf |  |  | 28202 | -0.773 | -0.2238 | Yes |
| 142 | 100330875 | gucy1a2 |  |  | 28291 | -0.784 | -0.2183 | Yes |
| 143 | 558552 | tubb1 |  |  | 28296 | -0.785 | -0.2102 | Yes |
| 144 | 767746 | zgc:153426 |  |  | 28740 | -0.866 | -0.2151 | Yes |
| 145 | 568406 | grm5a |  |  | 28855 | -0.888 | -0.2094 | Yes |
| 146 | 445289 | kras |  |  | 29269 | -0.994 | -0.2120 | Yes |
| 147 | 573122 | tuba1c |  |  | 29368 | -1.020 | -0.2044 | Yes |
| 148 | 572372 | gjd1a |  |  | 29472 | -1.053 | -0.1966 | Yes |
| 149 | 556286 | sos1 |  |  | 29488 | -1.060 | -0.1859 | Yes |
| 150 | 378480 | pdgfaa |  |  | 29776 | -1.156 | -0.1828 | Yes |
| 151 | 100006675\_1 | prkcba |  |  | 30078 | -1.276 | -0.1789 | Yes |
| 152 | 110438932 | LOC110438932 |  |  | 30733 | -1.709 | -0.1816 | Yes |
| 153 | 406811\_1 | zgc:55461 |  |  | 30849 | -1.809 | -0.1663 | Yes |
| 154 | 100000981\_2 | htr2cl1 |  |  | 30877 | -1.840 | -0.1478 | Yes |
| 155 | novel.946 | - |  |  | 31344 | -2.513 | -0.1362 | Yes |
| 156 | 386856\_1 | pdgfra |  |  | 31415 | -2.695 | -0.1101 | Yes |
| 157 | 571431 | LOC571431 |  |  | 31422 | -2.716 | -0.0818 | Yes |
| 158 | 566430\_1 | prkg1b |  |  | 31454 | -2.782 | -0.0536 | Yes |
| 159 | 560564\_2 | pdgfc |  |  | 31473 | -2.838 | -0.0244 | Yes |
| 160 | 323509 | gnaia |  |  | 31673 | -3.580 | 0.0068 | Yes |
Table: GSEA details [plain text format]

  

Fig 2: GAP\_JUNCTION(DRE04540)      
 Blue-Pink O' Gram in the Space of the Analyzed GeneSet

  

Fig 3: GAP\_JUNCTION(DRE04540): Random ES distribution      
 Gene set null distribution of ES for **GAP\_JUNCTION(DRE04540)**
